# Cell wall glycosyltransferase of *Streptococcus mutans* impacts its dissemination to murine organs

**DOI:** 10.1128/iai.00097-24

**Published:** 2025-02-20

**Authors:** Tomomi Hashizume-Takizawa, Taiki Ando, Ayaka Urakawa, Kazuhiro Aoki, Hedenobu Senpuku

**Affiliations:** 1 Department of Microbiology and Immunology, Nihon University School of Dentistry at Matsudo 47709 , Matsudo, Chiba, Japan; 2 Department of Basic Oral Health Engineering, Graduate School of Medical and Dental Sciences, Tokyo Medical and Dental University, Tokyo, Japan; 3 Department of Bacteriology I, National Institute of Infectious Diseases, Tokyo, Japan; University of Illinois Chicago, Chicago, Illinois, USA

**Keywords:** *Streptococcus mutans*, glycosyltransferase, glucosyltransferase, systemic challenge

## Abstract

*Streptococcus mutans*, a cariogenic bacterium in humans, is associated with systemic disorders. Its cariogenic factors include glucosyltransferases (GTFs) and the glycosyltransferase rhamnose–glucose polysaccharide I (RgpI), which is involved in cell wall synthesis. However, the potential roles of these enzymes in systemic disorders remain unclear. We constructed a luciferase-tagged *S. mutans* UA159 mutant strain that lacked *rgpI* to explore the involvement of this enzyme in the systemic pathogenicity of *S. mutans*. We also employed the luciferase-tagged *S. mutans* UA159 variant, which exhibited reduced GTF production and therefore had a low glucan synthesis ability. We intravenously inoculated these luciferase-tagged mutants and parent strains into 12-week-old male BALB/c mice to evaluate their distribution to organs. Strong luminescence was noted in the spleen and kidneys, indicating that *S. mutans* was disseminated to these organs. Several organs collected from mice inoculated with the luciferase-tagged parent strain emitted a signal, and inflammatory cytokine production was detected in the blood. The luminescence intensity was lower in the kidneys of mice challenged with the mutant strain, which has a low glucan synthesis ability. Conversely, challenge with the *rgpI* deletion mutant strain resulted in the lowest number of luminescent organs, with a lower intensity and attenuated inflammation. Furthermore, all the mice inoculated with the *rgpI* deletion mutant strain survived, whereas not all the mice inoculated with the parent strain survived. Collectively, these results suggest that RgpI is involved in the systemic pathogenicity of *S. mutans* UA159.

## INTRODUCTION

*Streptococcus mutans*, a gram-positive facultative anaerobic bacterium, is a major pathogen that causes dental caries in humans and is associated with systemic disorders, including infective endocarditis (1). Various pathogenic factors are involved in caries development, the most important of which are glucosyltransferases (GTFs) (2). *S. mutans* produces three types of GTFs (GTF-B, GTF-C, and GTF-D), which synthesize glucan from sucrose. Glucans are the main constituents of extracellular polysaccharides that are crucial for biofilm formation. Dental biofilms contribute to caries development in the oral cavity (3). Previous studies have shown that GTFs stimulate the production of inflammatory cytokines, including interleukin (IL)-6 and tumor necrosis factor alpha (TNF-α), from human T cells and endothelial cells (4, 5). When rats are intravenously injected with *S. mutans* GS-5 lacking GTF expression, the production of IL-6 in the blood and aortic valves with surrounding tissues is reduced compared with that induced by the challenge with wild-type *S. mutans* (4, 6). These studies suggest that the GTFs produced by *S. mutans* may act as inflammatory agents, potentially leading to virulence at extraoral sites. We previously demonstrated that the nasal immunization of mice with membrane vesicles containing GTFs released from *S. mutans* induced GTF-specific antibody responses (7). These findings suggest that GTFs are immunogenic. Thus, further investigations are needed to elucidate the systemic effects of GTFs.

The cell wall of gram-positive *S. mutans* is composed of a thick peptidoglycan layer and rhamnose-glucose polysaccharides (RGPs) that are covalently linked to a peptidoglycan, with RGP structures found uniquely in *S. mutans*. The RGP consists of a core polyrhamnose backbone and glucose side chain. *S. mutans* is categorized into serotypes *c*, *e*, *f*, and *k*, depending on the serotype-specific linkage of glucose side chains to the polyrhamnose main chain or the lack of glucose residues. The most prevalent serotype detected in the oral cavity is *c*, regardless of the presence or absence of caries (8, 9). Serotype *c* was most frequently detected from oral cavities of patients with the highest caries activity group (10). Thus, this serotype may be the most prevalent strain for caries development. The RGP structure of serotype *c* is characterized by an α-1,2 linkage of glucose side chains to a polyrhamnose backbone. Among the *rgp* genes involved in RGP synthesis, *rgpA*, *B*, and *F* contribute to the biosynthesis of rhamnose polymers, whereas *rgpE*, *H*, and *I*, which encode glycosyltransferases, contribute to the linkage of the glucose side chains to the polyrhamnose backbone (11, 12). RGPs stimulate human monocytes to secrete TNF-α (13). RGPs are involved in resistance to external stress, including oxidative stress, and, therefore, the pathogenicity of *S. mutans*. These stress functions may depend on the presence of the rhamnose backbone but not the glucose side chains, as the deletion in *rgpE* mutant strains persists under stress (12, 14). The loss of *rgpF* in *S. mutans* significantly reduces its virulence in a *Galleria mellonella* infection model (14). However, the glucose side chain might not branch in the *rgpF* deletion mutant strain, as branching may occur after the assembly of the polyrhamnose backbone. Thus, whether the attenuation of the virulence of *S. mutans* is attributable to the lack of a glucose side chain is unclear. In this context, previous studies have shown that an *rgpE* deletion, which lacks glycosidic residues, results in altered morphogenesis of *S. mutans* (12). In addition to the *rgpA* deletion mutant, which lacks the entire structure of RGP, the *rgpE* deletion mutant which lacks glycosidic residues has a minor effect on the development of infective endocarditis in a rabbit model (15). These studies suggest that the glycosylation of rhamnose polymers in the cell wall may also play a role in the pathogenicity of *S. mutans* in systemic disorders. *S. mutans* lacking *rgpI* (*SMU833*) has a reduced ability to form sucrose-dependent biofilms due to decreased GTF-B and GTF-C production, which is associated with diminished cariogenicity (16). Because GTFs may cause systemic inflammation, RgpI may also contribute to systemic disorders.

Serotype *k* strains of *S. mutans* are characterized by the lack of a glucose side chain in the rhamnose polymer (11). This serotype is highly pathogenic because it harbors a collagen-binding protein that interacts with the collagen layer of host cells, inhibiting platelet aggregation. A study showed that the collagen-binding protein expressed in this serotype is associated with hemorrhagic stroke and infective endocarditis (17, 18). However, the most prevalent strain in the human oral cavity is serotype *c*, and most such strains do not possess these proteins (19). Serotype *c* has been detected in heart valves and atherosclerotic plaques from patients with cardiovascular diseases, and inflammation is involved in its pathogenesis (20, 21). Thus, exploring the influence of this serotype on inflammation-related systemic disorders is essential.

Various methods are available for the detection of bacteria from human samples or murine organs after experimental inoculation (22, 23). PCR can be used to detect *S. mutans*-specific DNA. Cultivation methods that employ a selective medium for *S. mutans* can detect live *S. mutans* colonies. In addition, a recent study reported that the distribution of *S. mutans* in organs after experimental inoculation in mice can be detected using bioluminescence techniques, which employ luciferase-tagged *S. mutans* (23). This bioluminescence technique represents a more convenient, simple, and rapid approach than PCR and cultivation methods for observing the presence of *S. mutans* in *in vivo* experiments.

In this study, we employed serotype *c S. mutans* UA159, which is a collagen-binding gene *cnm* and *cbm*-deficient strain (24, 25). We constructed a *Renilla reniformis*-derived green luciferase-tagged *S. mutans* UA159 strain with a deletion in *rgpI* to elucidate the role of RgpI in systemic infection. A spontaneous mutant that was previously identified in the luciferase-tagged *S. mutans* strain UA159 results in reduced glucan synthesis (26, 27). Hence, this luciferase-tagged mutant strain was employed to assess the contribution of GTFs to the dissemination of the *S. mutans* to various organs. Luciferase-tagged *S. mutans* strains, including parent strains and mutants, were intravenously inoculated into mice, and the organs harboring the bacteria were detected using the bioluminescence approach. The inflammatory status and induction of antibody responses in inoculated mice were assessed to clarify the harmful effects of RgpI and GTFs on the systemic compartment.

## MATERIALS AND METHODS

### Bacterial strains and culture conditions

Table 1 presents the bacterial strains used in this study. Recombinant *S. mutans* strains were maintained and grown in brain heart infusion (BHI) broth (Becton, Dickinson, and Company, Franklin Lakes, NJ) or Mitis Salivarius (MS) agar (Becton, Dickinson, and Company) supplemented with erythromycin (10 µg/mL) with or without kanamycin (800 µg/mL) at 37°C in a 5% CO_2_ aerobic atmosphere (Gas pack: Mitsubishi Gas Chemical Co., Inc., Tokyo, Japan).

**TABLE 1:** Bacterial strains

Strains	Description	Reference
*S. mutans* UA159		
Δ*rgpI* (SMU833^-^)	*rgpl*::*erm*^r^	Nagasawa et al. (28)
Luciferase-tagged *S. mutans* UA159		
WT	*renG*, *erm*^r^	Urakawa et al. (26)
GSM	*renG*, *erm*^r^, low glucan synthesis mutant	Urakawa et al. (26)
Δ*rgpI*		
ΔRgpI	*renG*, *rgpl*::e*rm*^r^, *km*^r^	This study

**Fig 1:**
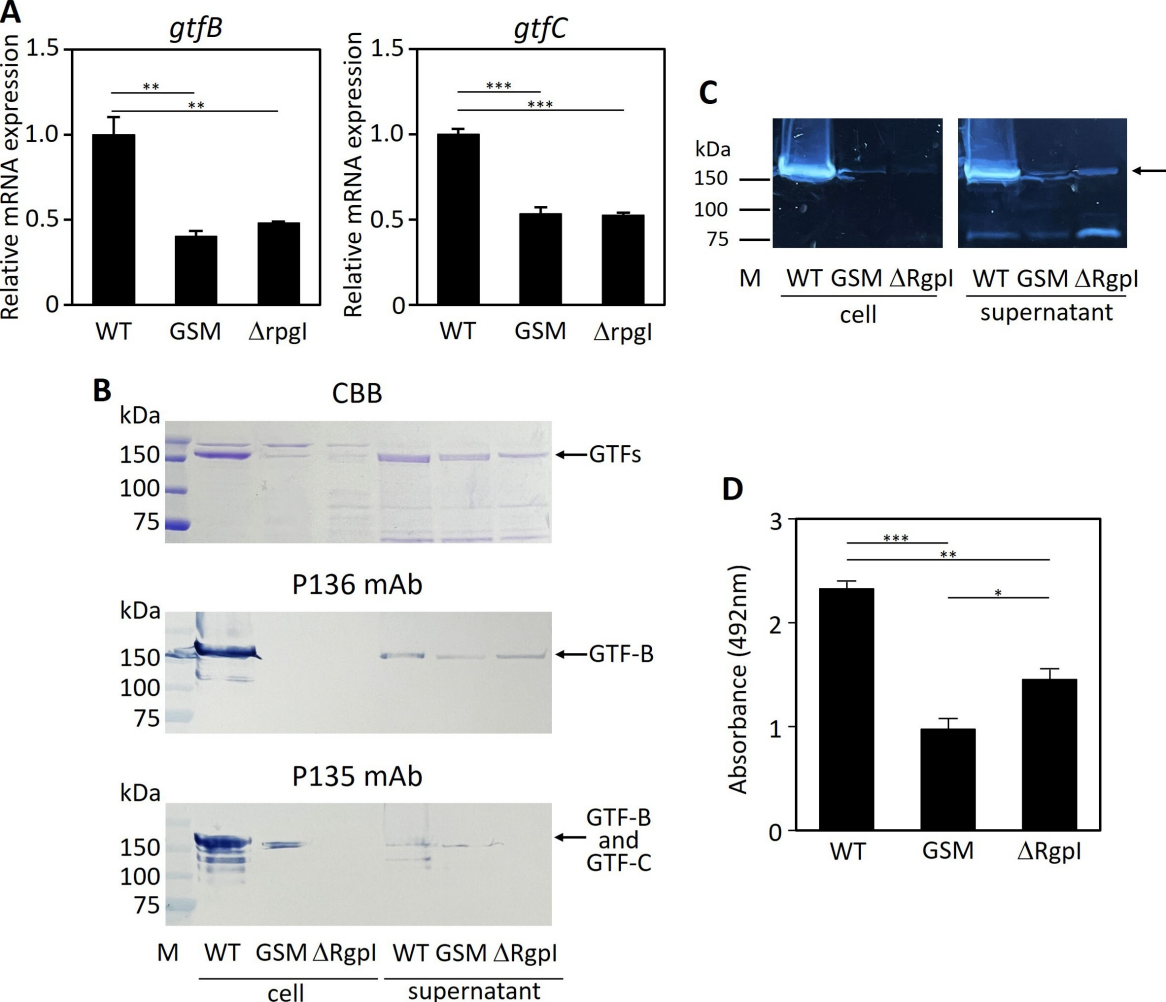
Comparison of *gtf* mRNA expressions and GTF contents and activity among the WT, GSM, and ΔRgpI strains. (**A**) *gtfB* and *gtfC* mRNA expressions in the WT, GSM, and ΔRgpI strains. mRNA levels were normalized to those of 16S rRNA and expressed as ratios relative to those of WT. (**B**) Proteins from whole-cell lysates and ethanol precipitates of supernatants were separated on 12.5% SDS-PAGE gels and stained with Coomassie Brilliant Blue (CBB). GTF bands were detected via Western blotting with anti-GTF-B (P136) and anti-GTF-C (P135), which slightly cross-reacted with GTF-B, monoclonal antibodies (mAbs) used as the primary antibodies. The arrows indicate GTF bands (approximately 150 kDa). Representative data from three independent experiments are shown in the images. (**C**) Zymography using buffer containing 3% sucrose. The arrow indicates the synthesized glucan. (**D**) Biofilm formation by the three strains in Trypticase soy broth supplemented with 0.25% sucrose was assayed by safranin staining. The data are presented as the means ± SEs. The experiments were repeated twice with similar results. The asterisks indicate a significant difference between each group, as determined by the Bonferroni correction. *P < 0.05, **P < 0.01, and ***P < 0.001. M, marker.

The luciferase-tagged *S. mutans* UA159 strain (designated WT in this study since this luciferase-tagged strain has a wild-type background) (Table 1) was constructed in our previous study (23, 26, 27). Briefly, the constructed lactate dehydrogenase (*ldh*)-luciferase gene fusion was amplified from the chromosome derived from luciferase-tagged *S. mutans* UA159 (the chromosome was kindly provided by Dr. Merritt [23]). The erythromycin resistance gene and the downstream *ldh* homologous fragment were amplified from the erythromycin resistance gene inserted at the multicloning site of pUC19 and the chromosome of *S. mutans* UA159. The three fragments (where one of the three fragments consisted of two genes) were ligated using overlap extension PCR with the primers Ldh up F/Ldh down R (Table 2). The assembled PCR amplicons were subsequently introduced into *S. mutans* UA159. Transformants were selected on MS agar supplemented with erythromycin (10 µg/mL). The light emission of the final transformants was confirmed (26). These final constructs included a spontaneous mutant, which formed a smooth colony in BHI agar with 0.25% sucrose (data not shown) (26, 29) and presented reduced *gtf* mRNA expressions that were associated with decreased GTF productions in cells. GTFs were detected in the culture supernatant at a low level in comparison with those of WT (Fig. 1A and B). An approximately 954–957 kb region encompassing the tandem *gtfB* (951112–955542) and *gtfC* (955738–960105) sequences of the reference genome (NC_004350.2) was deleted in this mutant (data not shown). Thus, this mutant presented reduced GTF activity, as shown by low glucan synthesis and biofilm formation ability (26, 27) (Fig. 1C and D). We employed a spontaneous mutant strain of luciferase-tagged *S. mutans* UA159 with diminished glucan synthesis and designated it glucan synthesis mutant (GSM) in this study (Table 1).

**TABLE 2:** Primers for mutant construction

Primer	Sequence*^a^*
Ldh up F	ccgctgagaaaggtaaatattagtgac
RenG dn R	gttttgaagaatgag**ttagagaatggctag**
Km(pUC19) F	**gatgtttagagaatg**acgcaaaccgcctct
Km(pUC19) R	ttagacgtcagg**tggcgacaataaaaatcc**
Ldh down F	**taagcccggcg**acaataaaaatcc
Ldh down R	ctgatgctattaaataagtgcgaaaacg

^a^ Additional sequence except sequences included in template DNA are indicated bold letters.

### Mice

Eleven-week-old male BALB/c mice (SLC Inc., Tokyo, Japan) were maintained under pathogen-free conditions in the experimental facility of the Nihon University School of Dentistry at Matsudo (Chiba, Japan).

### Construction of luciferase-tagged *S. mutans* Δ*rgpI*

**Fig 2:**
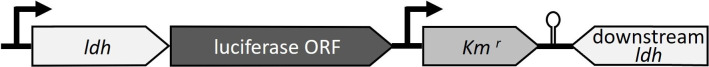
Sequences of constructs consisting of the four gene fragments. The luciferase open reading frames located immediately downstream of the *ldh* stop codons were subsequently cloned to create artificial two-gene operons. The luciferase cassette contained a copy of the *ldh* Shine-Dalgarno sequence to ensure its efficient translation.

The primers used to construct the luciferase-tagged *S. mutans* UA159 Δ*rgpI* deletion strain are listed in Table 2. We designated this constructed strain ΔRgpI in this study since this luciferase-tagged strain has an *rgpI* deletion background. ΔRgpI was generated using a four-piece (where one of four fragments consisted of two genes) overlap extension PCR ligation strategy, as described previously, with some modifications (23). Since the *S. mutans* UA159 Δ*rgpI* strain has an erythromycin resistance gene, kanamycin was employed as a selection marker. The kanamycin resistance gene was amplified from the gene inserted at the multicloning site of pUC19. The assembled PCR amplicons (Fig. 2) were subsequently introduced into the *S. mutans* UA159 Δ*rgpI* strain (28). Transformants were selected on MS agar supplemented with erythromycin (10 µg/mL) and kanamycin (800 µg/mL). The insertion of luciferase gene into the genomic DNA of the *S. mutans* Δ*rgpI* strain was confirmed by PCR.

### Recombinant bacterial challenge experiment

One week after arrival, the mice were randomly divided into four groups (*n* = 4). Overnight cultures of WT, GSM, and ΔRgpI were centrifuged (2,380 × *g*, 15 min, 4°C, TOMY LX-121, TS-36 swinging bucket rotor) and resuspended in phosphate-buffered saline (PBS) to achieve an optical density (OD) of 5 or 10 at 600 nm. The mice were intravenously inoculated with 200 µL of PBS or 200 µL of inocula of recombinant *S. mutans*. The inoculation was performed only once or three or five times per week over a total of 3 weeks. Two weeks after the final bacterial challenge, blood samples were collected, and the mice were euthanized via CO_2_ inhalation to isolate organ samples.

### Bioluminescence assays of the distribution of recombinant *S. mutans* in organs

The brain, cervical lymph nodes, salivary glands, heart, lungs, spleen, and kidneys isolated from the mice were homogenized individually in 2 mL of PBS using a TissueRuptor (Qiagen, Hilden, Germany) in repeated experiments. The homogenate was centrifuged at 500 × *g* and 4°C for 5 min to remove the debris. The supernatant was centrifuged again at 2,380 × *g* and 4°C for 13 min to collect the precipitated bacterial cells. Then, 500 µL of the luciferin substrate solution (diluted 5,000 times; ViviRen; Promega, Madison, WI) was added to each tube and incubated for 20 min at room temperature. Subsequently, 100 µL of each solution was dispensed into a 96-well black plate, and bioluminescence was measured using a luminometer (ARVO multilabel plate reader; Perkin Elmer, Waltham, MA). The count per second (cps) values were reported as the cps minus the corresponding values of the PBS-treated control. Aliquots of WT cultures were used as positive controls, and the cps values of the assay plates other than the wells were used as negative controls for standardizing bioluminescence.

### Counting bacterial colony-forming units (CFUs)

The CFUs of recombinant *S. mutans* distributed in organs were detected using MS agar plates supplemented with erythromycin. We used a bacterial suspension in the luciferin substrate solution, which was prepared as described previously. Fifty microliters of each suspension was spread onto erythromycin-supplemented MS agar plates. After 48 h of incubation, CFUs were analyzed. One of the colonies was cultured overnight in BHI broth supplemented with erythromycin. The precipitate was subsequently collected by centrifugation (2,380 × *g*, 4°C, 15 min) and suspended in the luciferin substrate solution to confirm luminescence.

### SDS-PAGE, Western blotting, and zymography

One milliliter of an overnight culture of the recombinant bacteria was centrifuged (2,380 × *g*, 4°C, 15 min) to separate the supernatants. The supernatants were mixed with an equal volume of chilled ethanol, followed by centrifugation (15,000 × *g*, 4°C, 20 min). The precipitates were collected and dissolved in distilled water. Sample buffer (0.06 M Tris-HCl, pH 6.8, 20% glycerol, 1% SDS, 1% 2-mercaptoethanol, and 0.0012% bromophenol blue) was added to these solutions and bacterial cells, which were then heated in boiling water for 5 min. The denatured samples were separated on 12.5% SDS-PAGE gels. The separated protein bands were visualized via Coomassie Brilliant Blue R-250 staining. For Western blot analyses, the separated proteins were transferred to Immobilon polyvinylidene fluoride membranes (Clear Blot Pt Membrane; ATTO, Tokyo, Japan). After blocking with 2% skim milk in Tris-buffered saline containing 0.05% Tween 20, monoclonal antibodies specific for GTF-B (P136) and GTF-C (P135), which cross-react with GTF-B slightly, were added as the primary antibodies (30), followed by a horseradish peroxidase (HRP)-conjugated anti-mouse IgG antibody (Invitrogen, Carlsbad, CA) as the secondary antibody to probe specific proteins. GTF-specific bands were detected by color development using 4-methoxy-1-naphthol (Sigma-Aldrich, St. Louis, MO) with hydrogen peroxide. For zymography, samples that had not been boiled were separated on a 12.5% SDS-PAGE gel, and the gel was subsequently incubated in an acetic acid buffer (pH 5.5) containing 3% sucrose at 37°C.

### Real-time reverse transcription polymerase chain reaction (RT-PCR)

Total RNA was purified from bacteria using a PureLink RNA Mini kit (Thermo Fisher Scientific, Waltham, MA) according to the manufacturer’s protocol. mRNA expression levels were quantified by using a PrimeScript RT Reagent kit, TB Green Premix Ex Taq II, and specific primers (Table 3) (16) (Takara Bio Inc., Shiga, Japan). mRNA expression levels were normalized to those of 16S rRNA. Quantitative RT-PCR was performed using a Thermal Cycler Dice Real Time System (TP850; Takara Bio Inc.).

**TABLE 3:** Primers for real-time RT-PCR

Primer	Sequence
16S rRNA F	cttaccaggtcttgacatccc
16S rRNA R	ccaacatctcacgacacgag
*gtfB* F	acactttcgggtggcttg
*gtfB* R	gcttagatgtcacttcggttg
*gtfC* F	caaaatggtattatggctgtcg
*gtfC* R	tgagtctctatcaaagtaacgcag

### Whole genome sequence

The chromosomal DNA of GSM was purified via a Wizard Genomic DNA Purification Kit (Promega, Madison, WI). The library was constructed via a ThruPLEX DNA-seq Kit and a Unique Dual Index Kit (Takara Bio Inc.). The quality of the sequence library was checked by an Agilent TapeStation (Agilent Technologies, Santa Clara, CA). Paired-end 150 bp sequencing was performed on a NovaSeq X Series 10B Reagent Kit and NovaSeq X Plus (Illumina, Inc., San Diego, CA), and the sequences were analyzed via NovaSeq X Series Control Software v.1.2.0, Real Time Analysis v.4.6.7, and BCL Convert v.4.3.6 (Illumina, Inc.). The resulting sequence reads were mapped to a reference genome (GCA_000007465).

### Analysis of cytokine production

The serum TNF-α and IL-6 levels were determined using enzyme-linked immunosorbent assays (ELISAs) (Thermo Fisher Scientific and R&D Systems Inc., Minneapolis, MN) according to the manufacturer’s protocol.

### Detection of recombinant *S. mutans*-specific IgG antibody responses

Recombinant GTF-B and GTF-C were purified from recombinant *Streptococcus milleri* KSB8 expressing *gtfB* and KSC43 expressing *gtfC* (31) as described previously (32). The overnight cultures of WT, GSM, and ΔRgpI strains were centrifuged (2,380 × *g*, 4°C, 15 min) and lyophilized. Antigen*-*specific antibody responses against the inoculated strain or the GTF-B and GTF-C proteins were determined using an ELISA, as described previously (33). Briefly, plates were coated with lyophilized bacterial cells, GTF-B, or GTF-C in PBS (bacterial cells; 10 µg/mL, GTF-B and GTF-C; 5 µg/mL) and blocked with 1% bovine serum albumin in PBS. Serial twofold dilutions of serum in PBS were added to the wells. Following a 4 h incubation at room temperature, the plates were washed with PBS supplemented with 0.05% Tween 20 (PBS-T). Then, HRP-labeled goat anti-mouse IgG antibodies (Southern Biotechnology Associates, Inc., Birmingham, AL) were added to the appropriate wells. After an overnight incubation at 4°C, the plates were washed with PBS-T, and 2,2’-azino-bis(3-ethylbenzothiazoline-6-sulfonic acid) with hydrogen peroxide was added for color development. End-point titers are reported as the reciprocal log_2_ of the last dilution that yielded an OD at 415 nm of 0.1 higher than the background after 15 min of incubation.

### Statistics

The data are presented as the means ± standard errors. Statistical analysis was performed using one-way analysis of variance (ANOVA) and repeated measures ANOVA with a *post hoc* Bonferroni correction, and Log-Rank Mantel-Cox test with SPSS software (version 29; IBM Corp., Armonk, NY). When the sample size was insufficient to perform a *post hoc* test, the results of the one-way ANOVA are shown on the *x*-axis. *P*-values <0.05 were considered indicative of statistical significance.

## RESULTS

### Construction of ΔRgpI

**Fig 3:**
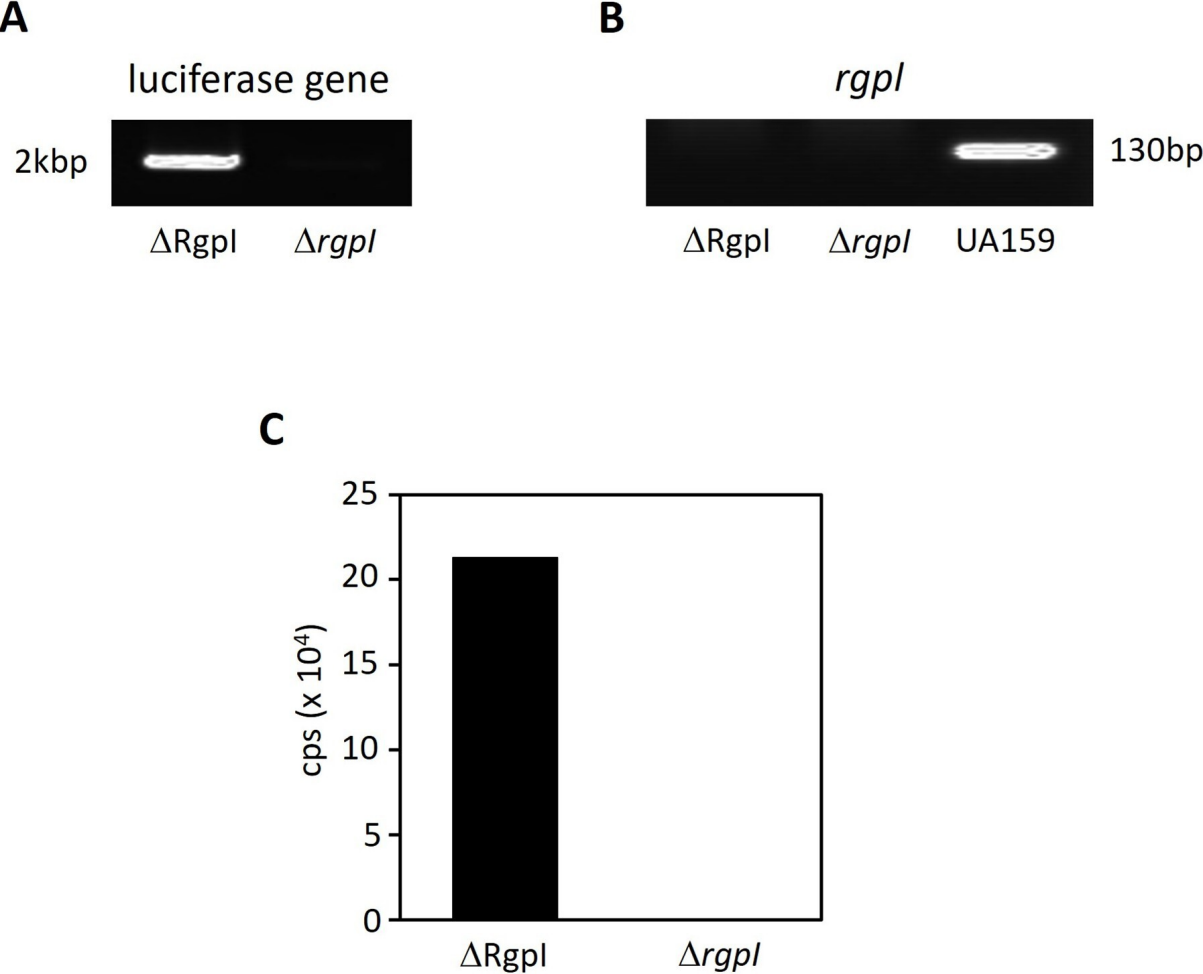
PCR identification of the luciferase gene and *rgpI* in the final transformant and confirmation of its light emission. Agarose gel electrophoresis of the PCR products of the luciferase gene (**A**) and *rgpI* (**B**) regions in the chromosome. (**C**) Light cps based on the luciferase activity of the final transformant toward luciferin as the substrate. The experiments were repeated twice with similar results.

A previous study constructed an *rgpI* deletion mutant strain of *S. mutans* UA159 by replacing the erythromycin resistance gene (*S. mutans* Δ*rgpI* strain) (28). Another study constructed a luciferase-tagged *S. mutans* UA159 strain in which luciferase gene was inserted immediately downstream of the stop codon of the *ldh* gene of the *S. mutans* UA159 chromosome (23). In this study, we constructed ΔRgpI to trace the distribution of this bacterium after intravenous inoculation into the mice. The fusion gene comprising *ldh* and luciferase gene, the kanamycin resistance gene as a selection marker, and the 1 kbp region downstream from the *ldh* stop codon were amplified using the primers listed in Table 2. These primers included artificially assigned, overlapping base sequences. The three fragments amplified were overlapped, and a series of fragments (Fig. 2) was introduced into the *S. mutans* UA159 Δ*rgpI* strain. The transformants were screened with MS agar supplemented with erythromycin and kanamycin. The insertion of luciferase gene and deletion of the *rgpI* gene from the chromosomal DNA of the final transformants and the light emission of the final transformants were confirmed (Fig. 3). ΔRgpI exhibited reduced *gtf* mRNA expressions and GTF secretions into culture supernatant. GTFs were detected in the cells at extremely low levels. ΔRgpI synthesized small amounts of glucan and biofilm (Fig. 1C and D).

### The WT strain causes high levels of lethality in mice

**Fig 4:**
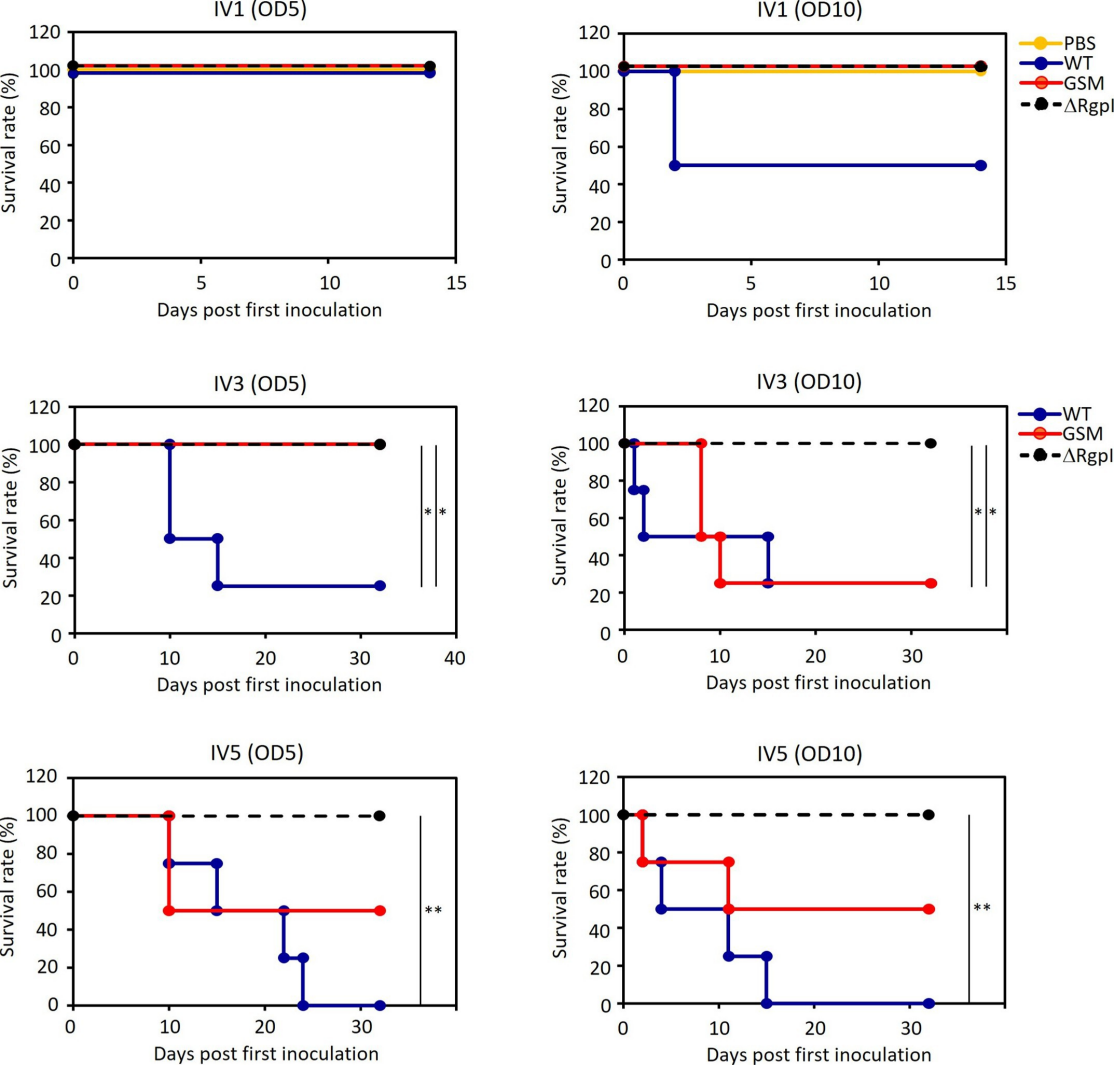
Kaplan-Meier curves for WT-, GSM-, or ΔRgpI-inoculated mice from the day of the first inoculation (*n* = 4 at the start of the experiment). The experiments were repeated twice with similar results. The asterisks indicate a significant difference between each group except the PBS-inoculated group, as determined by the Log-Rank Mantel-Cox test. *P < 0.05 and **P < 0.01. IV1, single-inoculation group; IV3 and IV5, three or five inoculations, respectively, per week for 3 weeks.

We explored the effects of *rgpI* deletion or mutation leading to reduced glucan synthesis on the dissemination of *S. mutans* UA159 to various systemic organs of mice. Thus, WT (26, 27), GSM (26, 27), and ΔRgpI were inoculated intravenously into mice at two doses; the OD_600_ of the inoculum was 5 and 10 (OD_600_ = 5 included approximately 5 × 10^8^ CFUs/mouse; OD_600_ = 10 included 1.4–4.5 × 10^9^ CFUs/mouse) for the induction of transient bacteremia, considering that no colonization was observed when the OD_600_ was adjusted to 1 (data not shown). The mice were inoculated with luciferase-tagged bacteria three or five times per week for a total of 3 weeks or only once, followed by 2 weeks of rearing. The number of surviving mice decreased upon challenge with WT or GSM strains as the number of inoculations increased (Fig. 4). Conversely, the mice inoculated with ΔRgpI exhibited 100% survival, irrespective of the number of inoculations and conditions (Fig. 4). The mortality of the mice inoculated with WT strain was greater than that of the mice challenged with the GSM strain. These results suggest that the reduction in glucan synthesis ability or incomplete formation of the cell wall due to the absence of *rgpI* might attenuate the harmful effects of *S. mutans* UA159 on extraoral organs.

### Detection of luciferase-tagged *S. mutans* mutant strains in mouse organs after intravenous inoculation

**Fig 5:**
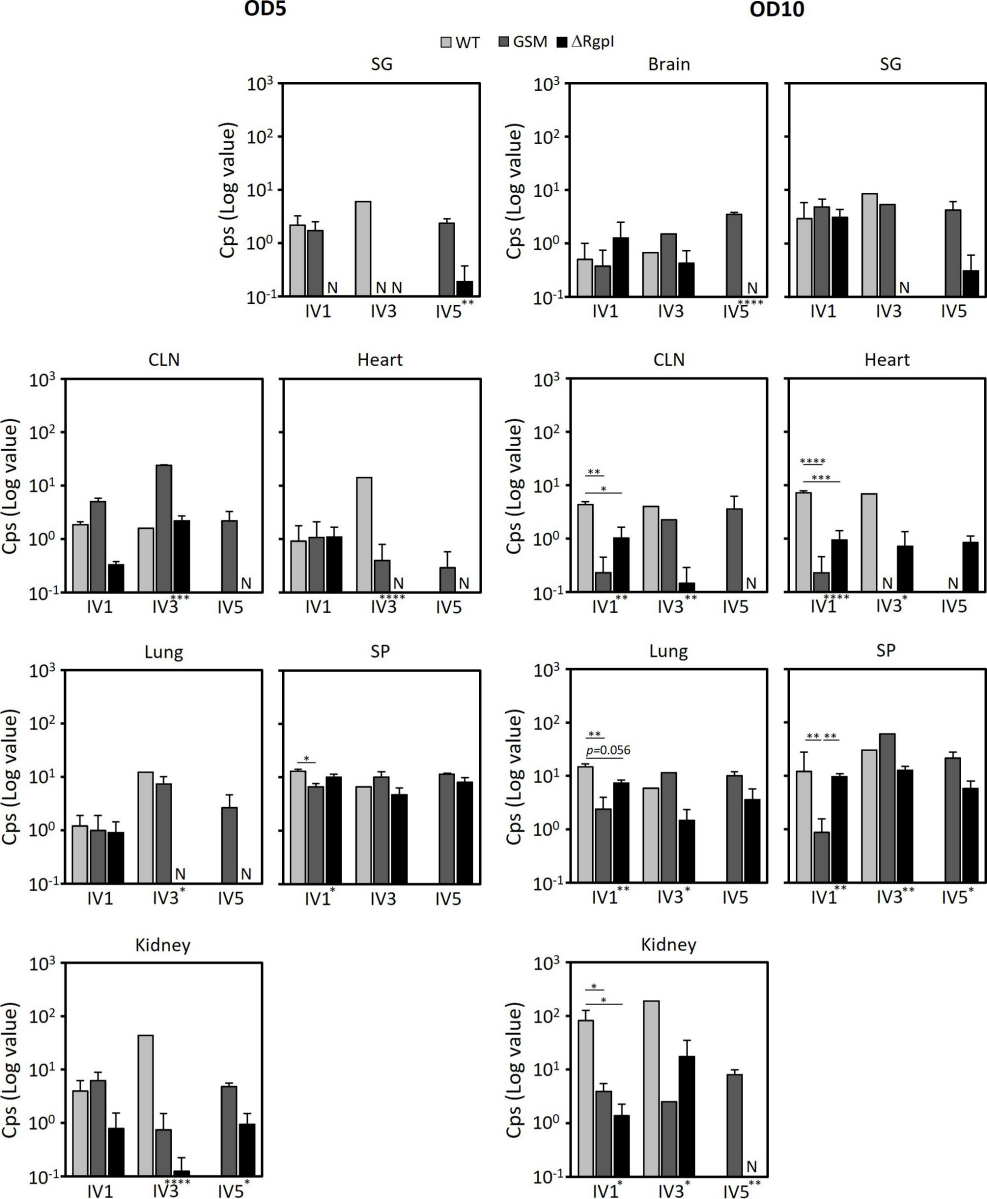
Detection of light produced from organ extracts isolated from mice inoculated with the WT, GSM, or ΔRgpI strain. The cps of various organ extracts was measured via a luminometer and is presented as the means of cps minus the corresponding value of the PBS-treated group ± SEs. The cps was not detected in the brain (OD5). The experiments were repeated twice with similar results. The asterisks on the *x*-axis indicate the results of one-way ANOVA. The asterisks on the bars indicate a significant difference between each group, as determined by *post hoc* Bonferroni correction. *P < 0.05, **P < 0.01, and ***P < 0.001. SG, salivary gland; CLN, cervical lymph node; SP, spleen; N, not detected.

Based on the findings that changes in the characteristics of *S. mutans* affect lethality in mice, we assessed the dissemination of various luciferase-tagged *S. mutans* mutant strains in mouse organs after intravenous inoculation. Luciferase-tagged *S. mutans* strains were detected by the luminescence of organ homogenates. Signals were detected in the salivary ground, cervical lymph nodes, heart, lungs, spleen, and kidneys of the mice injected with the WT strain (Fig. 5). Among them, the signals were strongest in the kidneys, and their intensities increased in a dose-dependent manner. In addition, this transformant led to the strongest signals of all transformants. The signals of the GSM strain tended to increase in relation to the dose in the cervical lymph nodes and lungs. The signal from spleens harboring the GSM strain tended to be stronger than spleens harboring the WT strain in the case of multiple inoculations. Conversely, in mice challenged with the ΔRgpI strain, signal was observed in the spleens, although most signals detected were around or below 10^0^, except for the lungs and kidneys, whose intensity tended to increase at ultrahigh doses (OD = 10). In summary, the bacterial distribution was greater for the WT strain than for the other two strains. ΔRgpI was cleared from the host, indicating that the localization ability of *S. mutans* UA159 was reduced by *rgpI* deletion.

**Fig 6:**
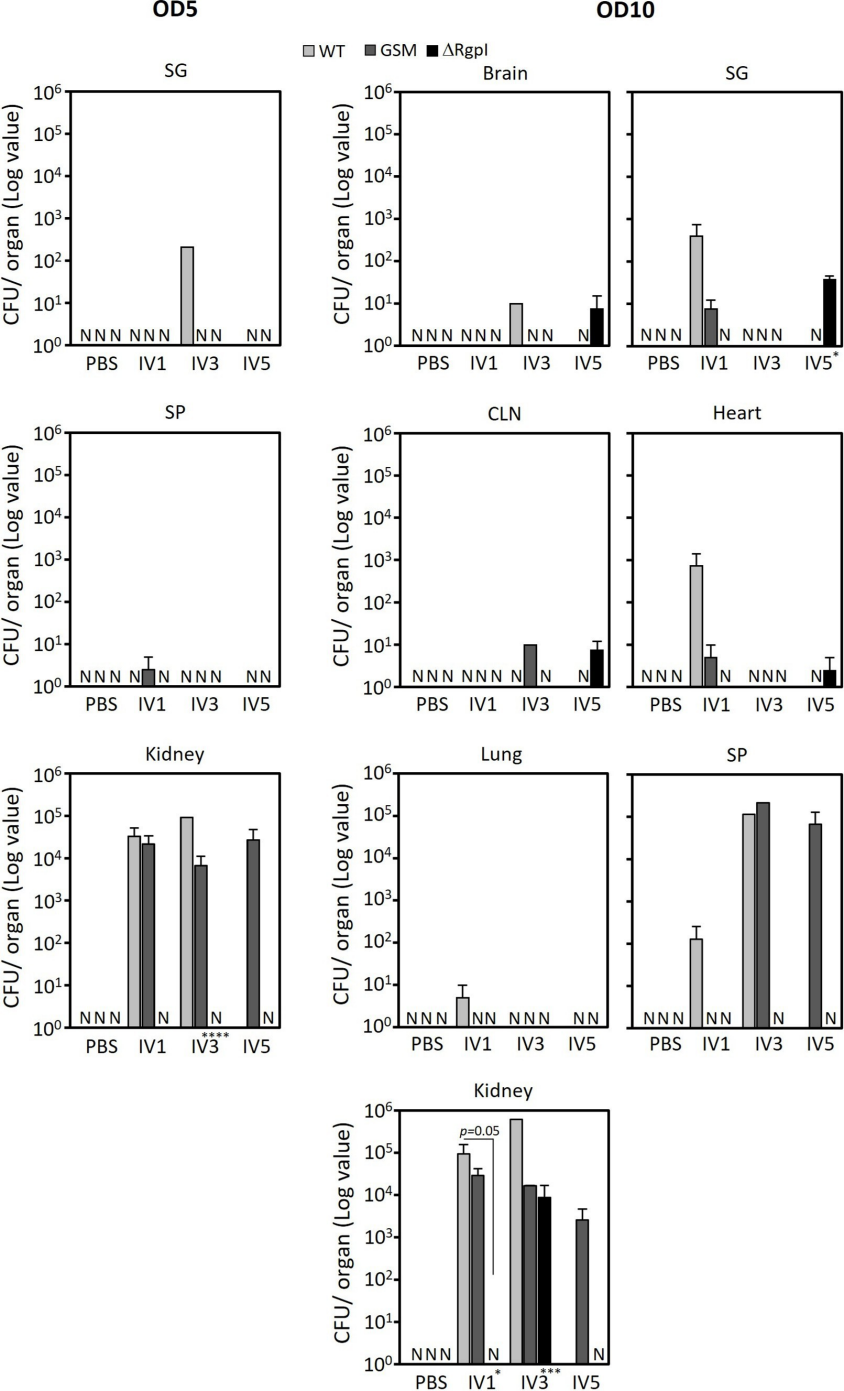
Dissemination of the WT, GSM, and ΔRgpI strains in various organs of mice inoculated with bacteria. Homogenates of various organs were spread onto MS agar supplemented with erythromycin. CFUs are presented as the means ± SEs. Colonies were not detected in the brain, cervical lymph nodes, heart, and lungs (OD5). The experiments were repeated twice, with similar results. The asterisks on the *x*-axis indicate the results of one-way ANOVA. The asterisks on the bars indicate a significant difference between each group, as determined by *post hoc* Bonferroni correction. *P < 0.05, **P < 0.01, ***P < 0.001, and ****P < 0.0001. N, not detected.

We analyzed the bacterial distribution to the organs again using cultivation methods to confirm the above results. Homogenates of organs removed from luciferase-tagged *S. mutans*-inoculated mice were plated on MS agar plates supplemented with erythromycin. Distinctive, blue, rough colonies were counted, and light emission from representative colonies was confirmed after culture of the colonies in BHI broth. Luciferase-tagged *S. mutans* colonies were again detected mainly in the heart, spleen, and kidneys but also in the salivary glands (Fig. 6). The number of colonies in the kidneys and spleen detected with this method was greater than that detected with the light-emitting method. Similar to the bioluminescent approach, the greatest number of colonies was detected from the mice inoculated with the WT strain. Conversely, ΔRgpI localized to murine organs at the lowest rates among the three strains, or no colonies were detected from the organs when the absorbance of the inoculum at 600 nm was adjusted to 5 (Fig. 6). According to the results from these two approaches, the WT strain preferentially localized to the kidney, and the localized bacterial load was diminished when glucan synthesis was impaired or when *rgpI* was deleted.

### *S. mutans* UA159 Δ*rgpI* exhibits low antigenicity without causing inflammation

**Fig 7:**
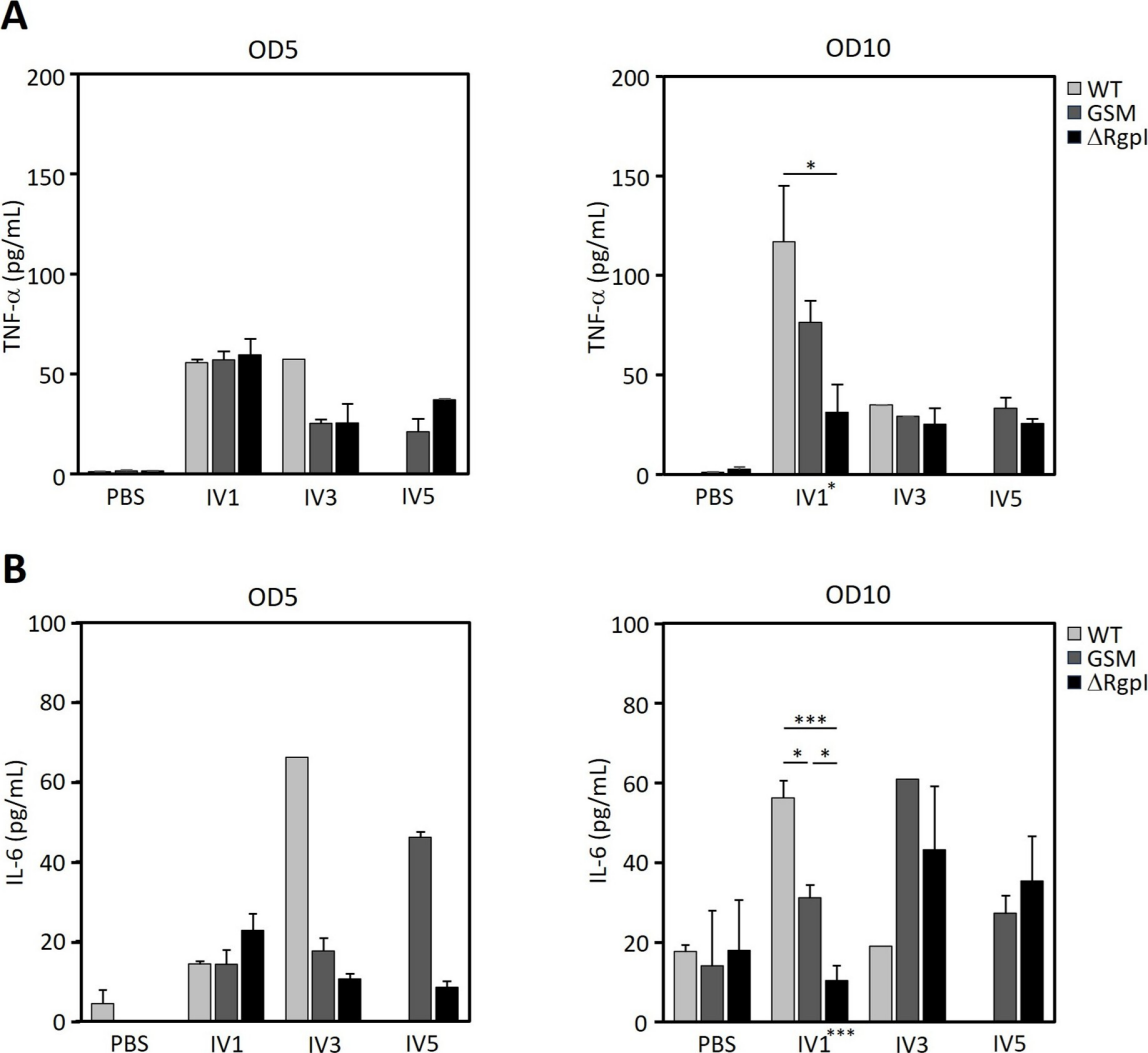
Inflammatory status in mice inoculated with luciferase-tagged *S. mutans* strains. Two weeks after the final inoculation, the serum was collected, and the serum TNF-α (**A**) and IL-6 (**B**) levels were analyzed using ELISAs. The data are presented as the means ± SEs. The asterisks on the *x*-axis indicate the results of one-way ANOVA. The asterisks on the bars indicate a significant difference between each group, as determined by *post hoc* Bonferroni correction. *P < 0.05, **P < 0.01, and ***P < 0.001.

**Fig 8:**
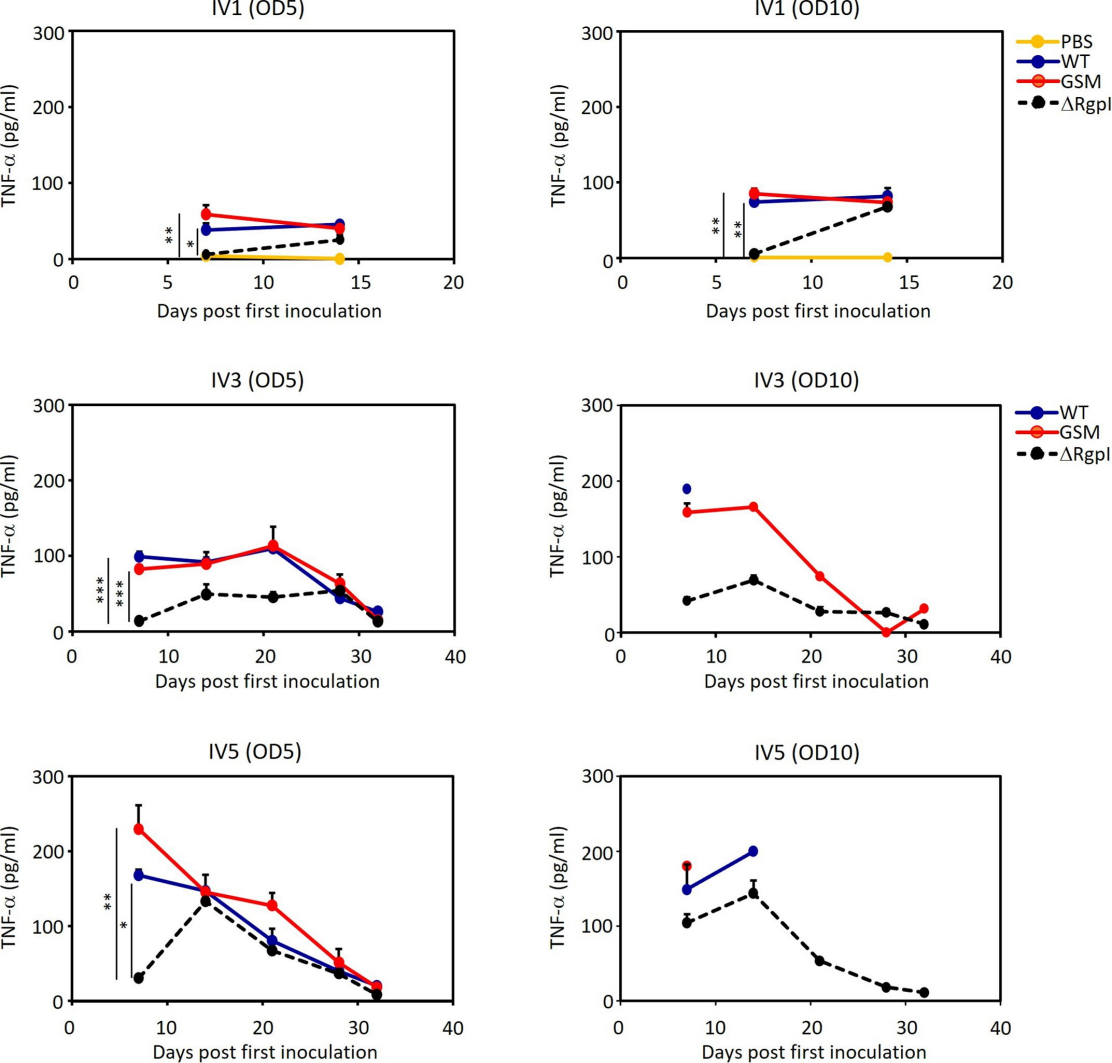
TNF-α production in the sera of mice inoculated with WT, GSM, or ΔRgpI. Serum was collected at weekly intervals, and TNF-α levels were analyzed via ELISA. The data are expressed as the means ± SEs. A significant difference between each group except the PBS-inoculated group was determined by repeated measures ANOVA with a *post hoc* Bonferroni correction. *P < 0.05, **P < 0.01, ***P < 0.001.

Inflammation is involved in the pathogenesis of systemic disease. Considering that lethality varied among the three mutant strains, we assessed whether the induction of inflammation and specific antibody responses in blood were altered among the three experimental groups. We assessed the levels of the inflammatory cytokines TNF-α and IL-6, since a previous study revealed that GTFs and RGP induce the production of these cytokines (4, 5, 13). The luciferase-tagged *S. mutans* strains induced the production of these cytokines to various degrees. Compared with ΔRgpI, WT and GSM elicited significantly higher levels of TNF-α production (Fig. 7A and 8). IL-6 production also tended to be higher in WT- and GSM-inoculated mice than in ΔRgpI-inoculated mice (Fig. 7B). The levels of TNF-α and IL-6 production were slightly different between WT- and GSM-inoculated animals throughout the experimental period (Fig. 7 and 8).

**Fig 9:**
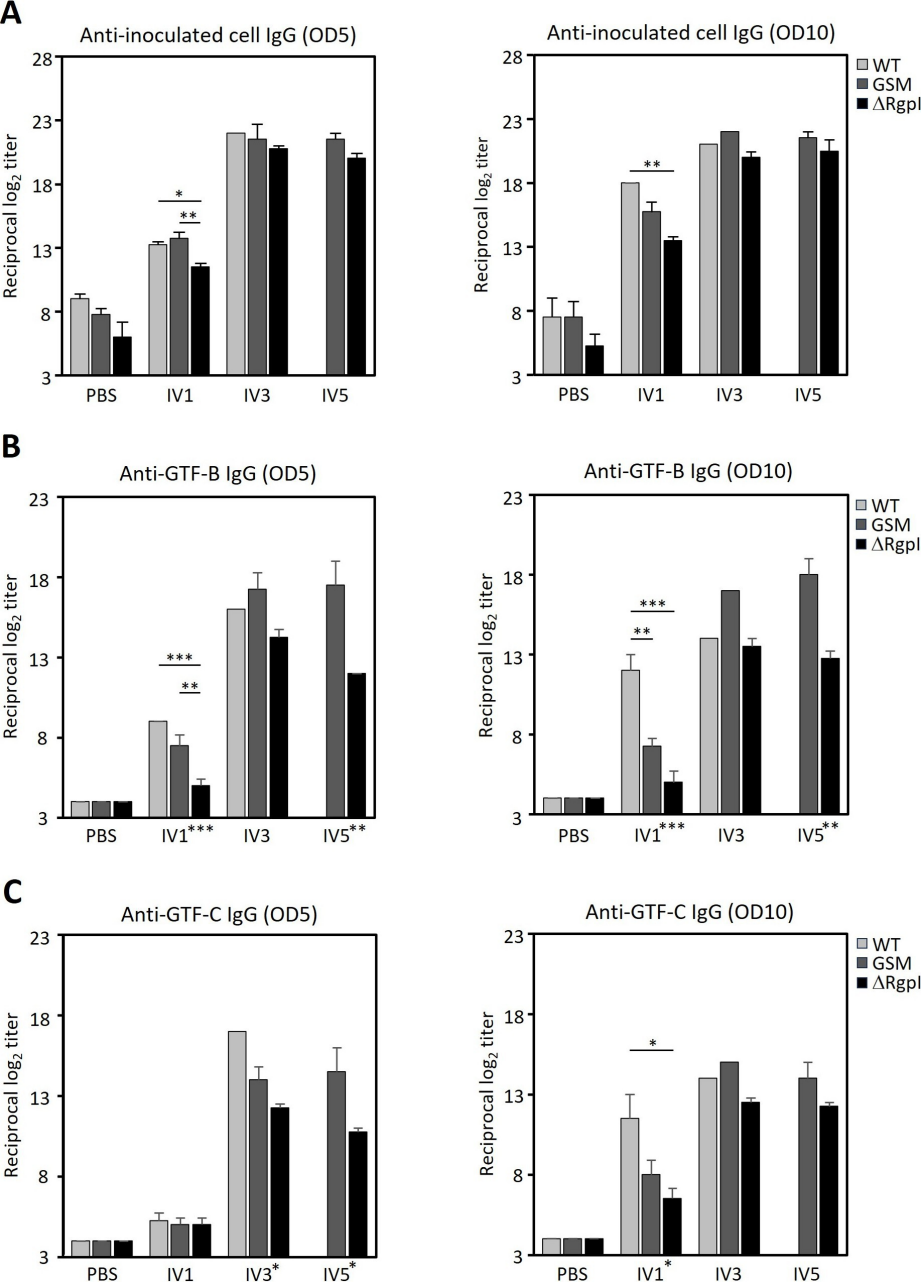
Inoculated *S mutans* strain-, GTF-B-, and GTF-C-specific IgG antibody responses in the sera of mice inoculated with the WT, GSM, or ΔRgpI strain. Two weeks after the final inoculation, the serum was collected, and the levels of specific IgG antibodies against the inoculated *S. mutans* strain (**A**), GTF-B (**B**), and GTF-C (**C**) were analyzed via ELISA. The data are presented as the means ± SEs. The experiments were repeated twice with similar results. The asterisks on the *x*-axis indicate the results of one-way ANOVA. The asterisks on the bars indicate a significant difference between each group, as determined by the Bonferroni correction. *P < 0.05, **P < 0.01, and ***P < 0.001.

**Fig 10:**
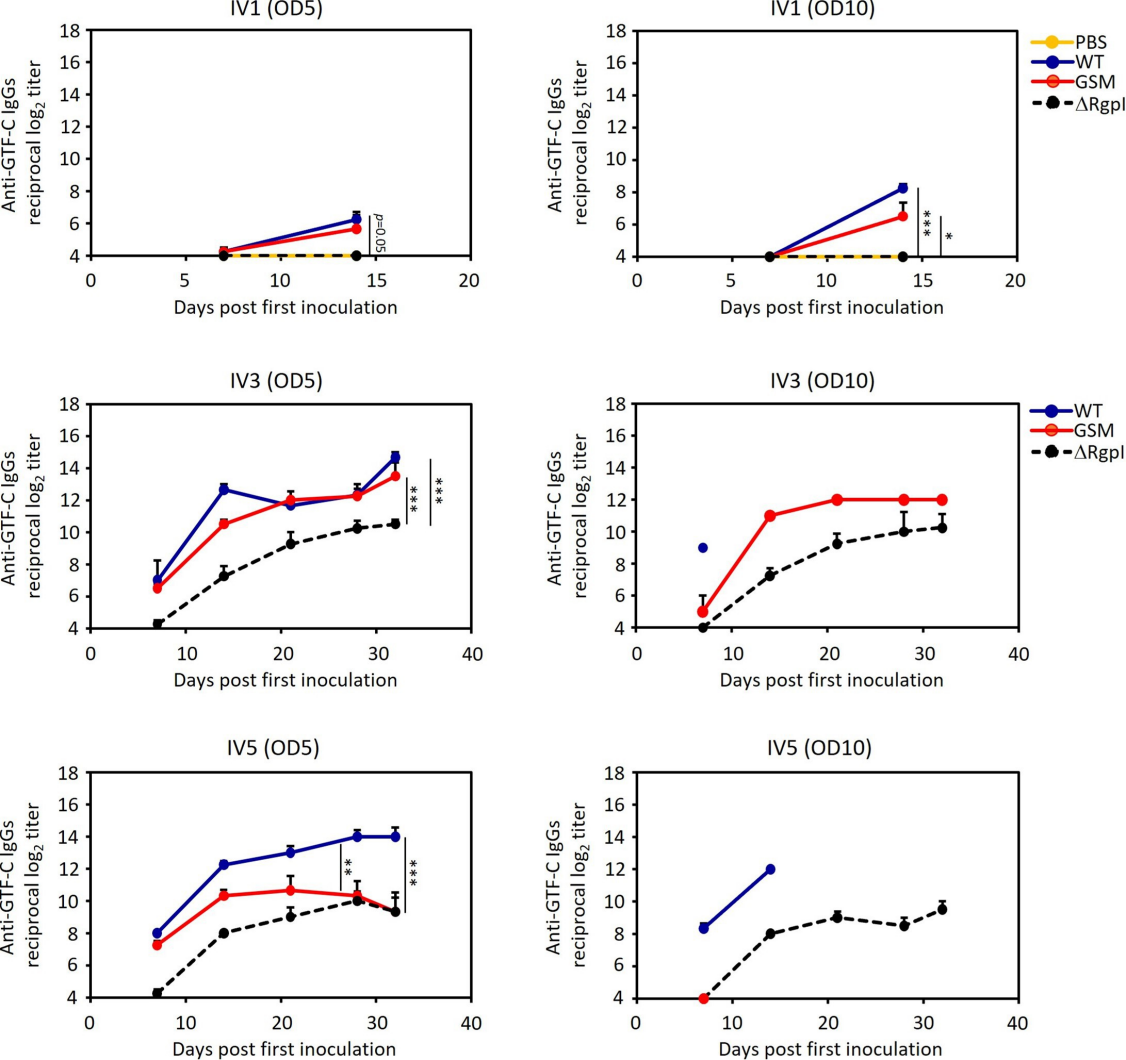
Serum GTF-C-specific IgG antibody responses in mice inoculated with WT, GSM, or ΔRgpI. Serum was collected at weekly intervals, and specific IgG antibody titers were analyzed via ELISA. The data are expressed as the means ± SEs. A significant difference between each group, apart from the PBS control, was determined via repeated measures ANOVA with a *post hoc* Bonferroni correction. *P < 0.05, **P < 0.01, ***P < 0.001.

**Fig 11:**
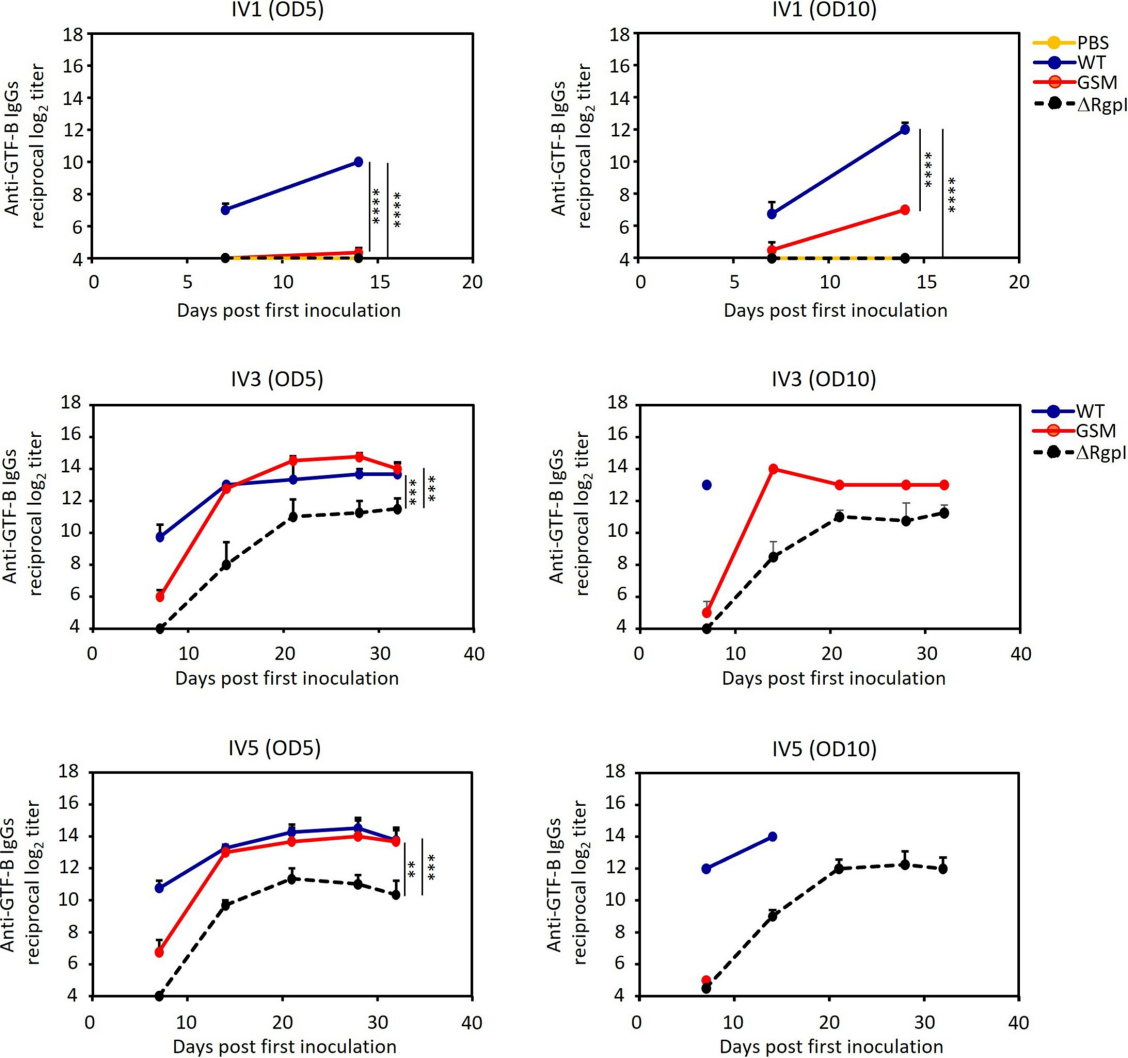
Serum GTF-B-specific IgG antibody responses in mice inoculated with WT, GSM, or ΔRgpI. Serum was collected at weekly intervals, and specific IgG antibody titers were analyzed via ELISA. The data are expressed as the means ± SEs. A significant difference between each group, apart from the PBS control, was determined via repeated measures ANOVA with a *post hoc* Bonferroni correction. *P < 0.05, **P < 0.01, ***P < 0.001.

Inoculated strain-specific IgG antibody responses were induced in the serum (Fig. 9A). A single inoculation of WT led to stronger antigenicity, with significantly higher levels of WT-specific IgGs in the serum of mice injected with both doses. Conversely, the inoculation of ΔRgpI resulted in lower levels of IgG against ΔRgpI in the serum. GSM induced intermediate IgG titers at an inoculum OD_600_ of 10 (Fig. 9A). High levels of inoculated strain-specific IgG responses were induced when the mice were inoculated with the recombinant strain multiple times. These levels were not significantly different among the groups. Similarly, compared with WT and GSM, ΔRgpI induced significantly lower levels of GTF-specific IgG responses (Fig. 9B, 9C, 10 and 11). The levels of GTF-specific IgGs in GSM-inoculated mice were almost equivalent to those in WT-inoculated mice after multiple inoculations (Fig. 10 and 11). These results indicate that GSM produces sufficient GTF-B/C to be immunogenic (34, 35) and that ΔRgpI produces smaller amounts of GTF-B and GTF-C with low immunogenicity. At least some of the changes in humoral immunity observed after a single exposure to the *S. mutans* strains are attributed to a decreased response against GTF-B and GTF-C.

These results indicate that the potential of *S. mutans* UA159 to stimulate innate and acquired immunity was decreased by the reduction in glucan synthesis and further decreased by the lack of glycosylation of the cell wall.

### The *S. mutans* UA159 Δ*rgpI* strain is characterized by growth in aggregates

Considering that the lethality, accumulation, and inflammatory effects of *S. mutans* UA159 were attenuated by reducing glucan synthesis and impairing cell wall structure due to the lack of *rgpI*, we compared the characteristics of the three strains. Their growth state after overnight culture was assessed.

**Fig 12:**
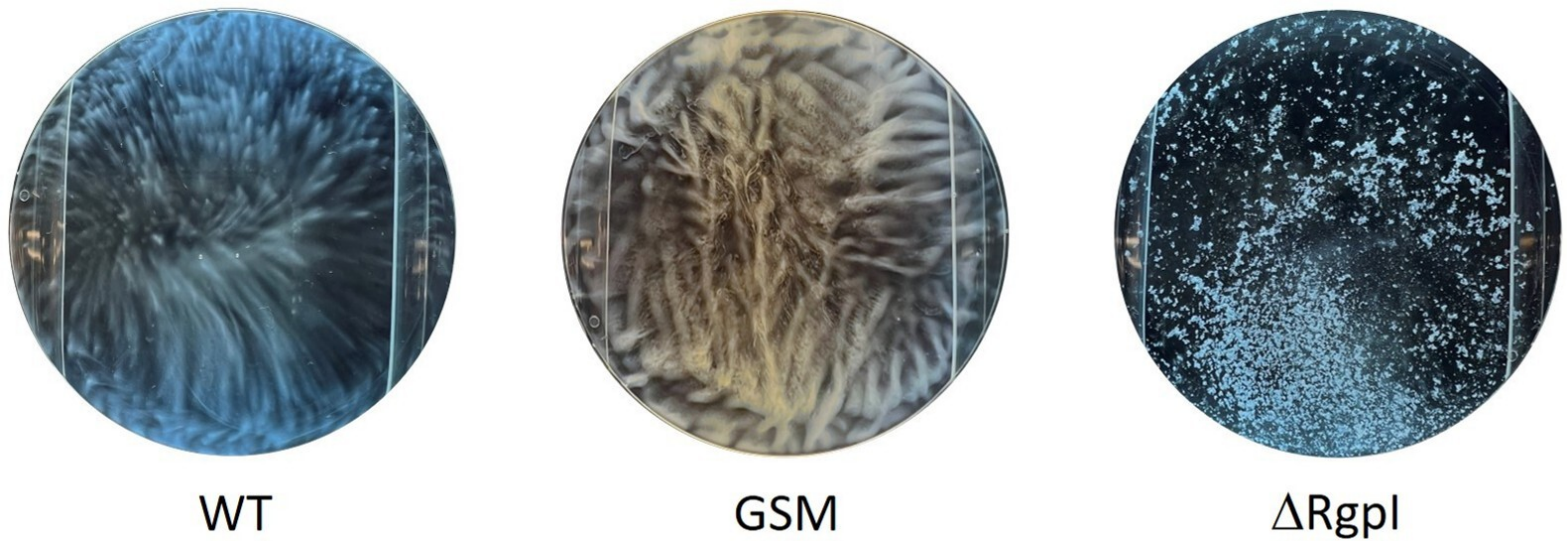
Comparison of the morphology of the WT, GSM, and ΔRgpI strains after overnight culture in BHI broth. Overnight precultures adjusted to 10^3^ CFUs/mL were cultured overnight. The experiment was repeated twice with similar results.

ΔRgpI formed aggregates in BHI broth after overnight culture. Conversely, WT and GSM exhibited normal growth, with an almost uniform cloudy liquid (Fig. 12). *S. mutans* UA159 bound to each other during growth in the absence of the glucose side chains of rhamnose polymers in the cell wall.

## DISCUSSION

We found that the lack of glycosylation of rhamnose polymers in cell wall polysaccharide resulted in attenuated harmful effects of *S. mutans* UA159 (serotype c), which exerted in the extraoral organs of mice. Mutation of *S. mutans* UA159, leading to reduced glucan synthesis ability, exhibited diminished localization ability. A previous study revealed that serotype *k S. mutans* lacks the glucose side chain of RGP structures and possesses highly pathogenic collagen-binding proteins that are involved in systemic disorders (17, 19). In this study, we showed that the glucose side chains and GTFs might be pathogenic factors of serotype *c S. mutans*, which has no collagen-binding proteins, for both oral infectious diseases and inflammation-related systemic disorders (16, 19). In this context, *S. mutans* UA159 does not contain genes encoding collagen-binding proteins Cnm and Cbm (24, 25), and harbors other adhesins, including the cell surface proteins P1, SapP, and the wall-associated antigen A, WapA (36). However, SpaP and WapA do not seem to be involved in the binding of *S. mutans* to collagen (37).

We detected significantly attenuated inflammatory responses in mice inoculated with ΔRgpI, which had defective glycosylation. Compared with those induced by the WT strain, lower levels of bacteria-specific IgG antibody responses were induced in the serum of mice inoculated with ΔRgpI. Therefore, the loss of glycosylation in the cell wall resulted in a reduced potential for the induction of inflammation and immunogenicity in these mice. Consistently, all the mice inoculated with ΔRgpI survived the experimental conditions, although we used very high doses as inocula. Our results are consistent with previous studies showing that TNF-α is produced by human monocytes stimulated with RGP (13) and that a serotype *c S. mutans* mutation that reduces the glucose content in the RGP is less likely to induce infective endocarditis in rats (15). This serotype naturally has RGP structures in which glucose side chains are bound to the polyrhamnose backbone by α-1,2 linkages (11). We found that the intravenous inoculation of the WT strain, which has intact RGP, led to high accumulation, inflammation, and lethality in mice under the experimental conditions (Fig. 4 and 5). The strongest luminescence was detected in the kidneys compared with the other organs. *S. mutans* might be trapped in the kidney due to the glomerular filtration barrier, which filters molecules less than approximately 50 kDa in the blood (38, 39). Previous studies reported the involvement of *S. mutans* in IgA nephropathy (40). Thus, IgG- or IgA-binding *S. mutans* immune complexes may be deposited in the glomeruli.

Our results indicated that all three strains accumulated in the spleen but that the luminescence of spleens harboring the GSM strain tended to be stronger than those of the spleens harboring the WT strain in the case of multiple inoculation. Antigens in the bloodstream are trapped by the spleen to induce antigen-specific immune responses (41). A previous study reported that an RGP-lacking serotype *c S. mutans* strain was more readily phagocytosed than the wild-type strain due to the increased hydrophobicity of the cell surface (42). Another study revealed that *S. mutans* UA159 lacking *gtfB* was more sensitive to hydrogen peroxide exposure than the wild-type strain (43). This suggests that GTF-B may play a role in bacterial tolerance to external stress. Collectively, these studies, together with our current study, suggest that RgpI deficiency might increase the likelihood phagocytosis, which may result in acceleration of bacterial clearance. Spontaneous mutations associated with reduced glucan synthesis might lead to increased recognition by phagocytes; thus, internalization by phagocytes might be increased.

We also found that *S. mutans* Δ*rgpI* strains grew as aggregates in BHI broth. In this context, a previous study revealed that the deletion of *rgpI* (*SMU833*) or *rgpH* in *S. mutans* UA159 increased the amount of extracellular DNA, resulting in increased extracellular DNA-dependent biofilm formation (16, 28). This biofilm formation was inhibited by DNase I treatment (28). The extracellular DNA released from *S. mutans* UA159 promotes adhesion among bacteria and enhances biofilm formation (44, 45). The present study and these previous studies suggest that the lack of glycosylation of the RGP in the cell wall might enhance cell aggregation by increasing the quantity of extracellular DNA. Thus, when serotype *c S. mutans* lacks glucose side chains in the cell wall, this bacterium may decrease its adhesion to host cells and the rate of localization due to bacterial aggregation, resulting in increased host clearance and reduced inflammation and lethality.

Glucans, which are components of extracellular polysaccharides, are converted from sucrose by glucan synthases, including GTFs produced by streptococci. GTFs are cariogenic factors of *S. mutans* (3). Interestingly, when the mutant strain of *S. mutans* UA159, characterized by low glucan synthesis ability, was intravenously inoculated into mice, reduced accumulation and the potential to induce bacteria-specific IgG antibody systemic responses were observed compared with that of the wild-type strain. In this context, an experimental infection study revealed that the survival rate of *Galleria mellonella* larvae was slightly higher after exposure to *S. mutans* UA159 lacking *gtfB* than to *S. mutans* UA159 (43). These findings suggest that GTF-B partially contributes to the harmful effects of *S. mutans* on the host during systemic challenge. The production of the inflammatory cytokines IL-6 and TNF-α by human umbilical vein endothelial cells and human peripheral blood-derived T cells increases in response to GTF-C stimulation (4, 5). In a previous study, when rats were intravenously inoculated with *S. mutans* GS-5 (serotype *c*) deficient in the expression of GTF-B, GTF-C, and GTF-D, serum IL-6 levels were decreased compared with those measured after challenge with the wild-type strain (4). On the other hand, our results showed that inflammatory cytokine production induced by the GSM strain was not different from that induced by the WT strain throughout the experimental period, although IL-6 and TNF-α productions tended to decrease slightly, which was more evident in the lower dosage groups. This effect may be masked in the higher dosage groups because of the decreased sample size at later timepoints. The GSM strain induced bacterial component-specific IgG responses at levels identical to those of the WT strain after multiple inoculations, even though lower responses were noted after a single injection. These findings support the low GTF production ability of this strain, but these GTFs are sufficient to be immunogenic and possibly cause inflammation. GSM exhibited a smooth-type colony (26) and did not aggregate after an overnight culture in BHI broth. In this context, a previous study reported that *S. mutans* strains lacking water-insoluble glucans exhibited a smooth-type colony (29) and retained GTF production with low GTF function due to recombination in *gtfB* and *gtfC* (34). Thus, the decreased functions of GTFs may not be responsible for the increase in extracellular DNA levels. Taken together, these findings suggest that, compared with ΔRgpI, GSM is presumed not to increase the amount of extracellular DNA due to its intact RGP structure, which may lead to a lack of aggregate formation and therefore stronger adherence to and accumulation in host cells. *RgpI* deficiency leads to reduced *gtf* expression, production of GTF proteins (16) and the induction of GTF-specific IgG responses. Because *rgpI* deficiency leads to an increase in the level of extracellular DNA, genomic DNA, including the sequences encoding GTFs, may be released extracellularly in some bacteria. The reduced pathogenicity of ΔRgpI may be attributed to diminished GTF production in addition to a lack of RgpI. Taken together, the previous and present studies suggest that GTF production may be responsible for the harmful effects of *S. mutans* on the host.

Our results revealed that the attenuation of the harmful effects observed in the GSM strain exhibiting reduced GTF production was further attenuated in the ΔRgpI strain, which lacked glycosylation and had lower GTF production. These findings suggest that the reduced virulence of the ΔRgpI strain may be attributed to a lack of glycosylation, reduced GTF production, and excessive extracellular DNA production (16).

We used bioluminescence methods to detect organs infected with *S. mutans*. A previous study constructed *R. reniformis*-derived green luciferase-tagged *S. mutans* and successfully detected oral bacteria, including *S. mutans*, using luciferase-luciferin systems (23). Luciferin is oxidized by luciferase expressed in *S. mutans*, resulting in light emission. Thus, we constructed a luciferase-tagged *S. mutans* UA159 Δ*rgpI* strain and performed tracing experiments after the inoculation of the strain into mice. We also detected luciferase-tagged *S. mutans* colonies from organs via cultivation methods without light emission. Despite the discrepancy between the two approaches, the bacterial colonization rates were consistent with the results obtained using bioluminescence methods. Both methods showed that *S. mutans* UA159 colonized organs at relatively high levels and that *S. mutans* UA159 Δ*rgpI* colonized them at relatively low levels. Thus, the distribution of luciferase-tagged *S. mutans* in murine organs was successfully detected using the luciferase-luciferin system.

In summary, the production of GTFs, which catalyze glucan synthesis, may contribute to the harmful effects of *S. mutans* on the systemic organs of the host. Furthermore, the glycosyltransferase RgpI, which plays crucial roles in the glycosylation of the polyrhamnose backbone of the RGP structure in the cell wall, may contribute to the pathogenicity of serotype *c S. mutans* in extraoral organs. In *S. mutans*, *rgpI* deletion resulted in reduced GTF production. This deletion had a more pronounced effect on attenuating the adverse effects of serotype *c S. mutans* on the host, possibly due to a reduction in bacterial adhesion to host cells. Thus, RgpI may be more important than GTFs for the virulence of serotype *c S. mutans* throughout the entire body.

## Data Availability

The raw sequence data of the GSM genome were deposited in the DNA Data Bank of Japan Sequence Read Archive under accession number DRR628311.
